# Brivaracetam population pharmacokinetics in children with epilepsy aged 1 month to 16 years

**DOI:** 10.1007/s00228-017-2230-6

**Published:** 2017-03-09

**Authors:** Rik Schoemaker, Janet R Wade, Armel Stockis

**Affiliations:** 1 SGS Exprimo, Mechelen, Belgium; 2 Occams, Malandolaan 10, 1187 HE Amstelveen, The Netherlands; 3 Clinical Pharmacology, UCB Pharma, Braine l’Alleud, Belgium

**Keywords:** Brivaracetam, Paediatrics, Epilepsy, Population pharmacokinetics, NONMEM

## Abstract

**Purpose:**

The aims of the study were to develop a population pharmacokinetic model of orally administered brivaracetam in paediatric patients and to provide dosing suggestions.

**Methods:**

Analysis included 600 brivaracetam plasma concentrations from a phase 2a study (NCT00422422; N01263) in 96 paediatric patients with epilepsy aged 1 month to 16 years, taking one to three concomitant antiepileptic drugs (AEDs). Pharmacokinetic analysis was performed using non-linear mixed effects modelling, and a stepwise covariate search was used to determine factors influencing brivaracetam clearance. Simulations were performed to investigate dosing regimens.

**Results:**

The final model consisted of first-order absorption, single compartment distribution and first-order elimination components with allometric scaling of clearance and volume using lean body weight and fixed allometric exponents. Co-administration with phenobarbital or carbamazepine was associated with a 29% (95%CI 17%/39%) and 32% (22%/42%) decrease in exposure, respectively. Co-administration with valproate was associated with an 11% (1%/23%) increase in exposure. Simulations demonstrated that the majority of children were predicted to have an exposure similar to that in adults, using an age-independent dosing regimen of 2.0 mg/kg bid with a maximum of 100 mg bid for body weight >50 kg.

**Conclusions:**

A paediatric dose adaptation of 2.0 mg/kg twice daily with a maximum of 100 mg twice daily for body weight >50 kg is predicted to ensure steady-state plasma concentrations in the same range as in adult patients receiving 100 mg twice daily (highest recommended dose). Data suggest no need to change brivaracetam dosing when used concomitantly with carbamazepine, phenobarbital or valproate.

**Electronic supplementary material:**

The online version of this article (doi:10.1007/s00228-017-2230-6) contains supplementary material, which is available to authorized users.

## Introduction

Seventy million people have epilepsy with 34–76 per 100,000 developing the condition every year [1]. The incidence varies greatly with age, with high rates occurring in childhood, falling to low levels in early adult life, but with a second peak in those aged over 65 years. Epilepsy affects about 4 to 6 out of 1000 children below the age of 20 years, and the overall annual incidence rates of epilepsy for all seizure types range between 45 and 86 out of 100,000 children. Despite the availability of new antiepileptic drugs (AEDs), more than 25% of paediatric patients have inadequate seizure control on currently available AEDs, or experience significant adverse drug effects [2]. There remains a need for potent AEDs with a positive benefit-risk profile in this population.

Brivaracetam is a selective, high-affinity synaptic vesicle protein 2A ligand [3] that was recently approved as adjunctive therapy in the treatment of focal (partial onset) seizures in patients 16 years of age and older with epilepsy [4–8]. Brivaracetam is rapidly and highly absorbed and peak plasma concentrations are generally reached within 2 h after dosing in fasting subjects [9, 10]. The disposition of brivaracetam is characterised by linear pharmacokinetics over a large range of doses (10 to 600 mg). Brivaracetam is eliminated primarily by metabolism, which is partially cytochrome P450 dependent. The three main metabolites are not pharmacologically active. Only a small fraction (up to 10%) of the dose is excreted as parent compound in the urine [11]. Brivaracetam is available as bioequivalent tablets, oral solution and intravenous injection [12, 13].

We report the results of non-linear mixed effects modelling of brivaracetam pharmacokinetics from a phase 2a trial in paediatric epilepsy patients (NCT00422422; N01263 [14]), including a covariate analysis and determination of paediatric dosing adaptations.

## Methods

Trial N01263 was conducted in accordance with the International Conference on Harmonization notes for Guidance on Good Clinical Practice and the Declaration of Helsinki. The study protocol was approved by institutional review boards at all study sites, and written informed consent was obtained from all parents or guardians before enrolment.

### Data

Trial N01263 was an open-label, single-arm, multicentre, fixed 3-step up-titration study evaluating the pharmacokinetics, safety, and efficacy of brivaracetam in children aged ≥1 month to <16 years. Brivaracetam oral solution was administered at weekly increasing doses of approximately 0.4 mg/kg bid, 0.8 mg/kg bid, and 1.6 mg/kg bid for subjects ≥8 years of age and 0.5 mg/kg bid, 1.0 mg/kg bid, and 2.0 mg/kg bid for subjects <8 years of age. The doses were to be capped at the adult doses of 25 mg bid, 50 mg bid and 100 mg bid, respectively, for body weight (WT) ≥50 kg.

The study planned to enrol 100 children (≥1 month to <16 years), recruited in approximately 50 sites, with localisation-related, generalised or undetermined focal or generalised epileptic syndrome, according to the International League Against Epilepsy classification. Patients had to be receiving one to three concomitant AEDs, except levetiracetam which was not allowed. Hepatic impairment was an exclusion criterion for entry in the clinical trial. Subjects completed a 1-week prospective baseline period, followed by a 3-week evaluation period with a weekly fixed 3-step up-titration based on age. On the last of 7 days of brivaracetam dosing at a scheduled dose level (i.e. at day 7, day 14, and day 21), two scheduled blood samples were drawn for plasma determination of brivaracetam and metabolites in one of three possible time brackets: early morning (one sample before and one between 1 and 2 h post morning dose), late morning (two samples between 2 and 6 h post morning dose, at least 2 h apart) or afternoon (two samples between 6 and 12 h post morning dose, at least 2 h apart), plus one optional blood sample at a later or intervening time. Only brivaracetam parent compound data were used for the population pharmacokinetics modelling. Metabolites data were used for simple estimations of relative exposures.

Estimated body surface area-normalised glomerular filtration rate (eGFR) was calculated using the Schwartz bedside equation [15, 16]. Lean body weight (LBW) was calculated from total body weight and body mass index (BMI) [17]. Post-conceptional age (PCA) was only considered relevant for patients below 3 years; for all other patients, and for patients where PCA was missing, PCA was calculated as post-natal age (years) + 0.75. A summary of the population characteristics and main covariates available for inclusion in the analysis is provided in the online supplementary material as Supplemental Table 1. The validated bioanalytical method is also available in the online supplementary material.

### Software

The analyses were performed using NONMEM Version 7.2.0 [18] software using the First Order Conditional Estimation with the interaction option (FOCE-I). 95% confidence intervals (95%CI) were calculated as the estimate ± 1.96 times the NONMEM-reported standard error for the estimate. An additional assessment of parameter uncertainty not relying on normality assumptions was obtained by bootstrapping the final model 1000 times [19]. Data were further processed using 64 bit R Version 2.15.2 software [20]. Simulations were performed using NONMEM and R. Stepwise covariate modelling (SCM, [21]) and bootstrapping was performed using Perl Speaks NONMEM (PsN) [22] version 3.4.2.

### Brivaracetam population pharmacokinetics model

The structural model was a one-compartment model with first order absorption and elimination together with allometrically scaled effects of body size on clearance (CL) and volume of distribution (V). The influence of body size was estimated using the following equation:

$$ {PAR}_i={\theta}_1\cdot {\left(\frac{COV_i}{POP_{COV}}\right)}^{\theta_2}\cdot {e}^{\eta_i} $$

where *θ*_1_ is the population value of the estimated pharmacokinetic parameter, *PARi* is the individual-specific parameter value for the i^th^ subject with the value of the covariate (*COVi*) scaled to a population typical value (*POP*_*COV*_). Both body weight (WT) and LBW were assessed as measures of body size. For WT, an adult population typical value of 70 kg and for LBW a typical value of 50 kg were used for *POP*_*COV*_ to allow easy comparison with reported adult values. The parameter *θ*_2_ is the scaling parameter for the weight range. If allometric scaling principles are applied, *θ*_2_ takes on specific (fixed) values for the influence of body size; these values are 0.75 for CL and 1 for V [23].

Although LBW is not a very convenient measure for dosing adaptations in clinical practice, it was recognised that it might prove superior in describing the underlying pharmacokinetic properties. However, dosing recommendations were developed based on simple clinically applicable rules (i.e. WT-based for specific age ranges).

Exponential models were used to describe the inter-individual variability (IIV) for the structural model parameters. IIV was calculated as the square root of the diagonal element in the omega matrix. Proportional and combined additive and proportional models were investigated to describe the residual variability. Proportional error model components were reported as coefficient of variation (CV).

Comparison between various potential models was based on a likelihood ratio test using the difference in the NONMEM-provided Objective Function Value (OFV) for two hierarchical competing models and where the number of degrees of freedom is equal to the difference in parameter numbers between the two models. For structural model updates, the more complex model was required to be associated with a *p* value less than 0.01.

The covariates tested in the model for their effects on brivaracetam clearance were age, PCA, sex, race, ethnicity (Hispanic/Latino or not), eGFR, carbamazepine co-administration (CBZ), phenobarbital or primidone co-administration (PB), valproate co-administration (VPA), inducer co-administration (presence of CBZ, phenytoin (PHT) or PB), non-AED CYP3A inhibitor co-administration, and non-AED CYP2C19 inhibitor co-administration. The effect of PHT was not assessed since only a single patient had this treatment at entry in the study. Categorical covariates were investigated in the SCM approach using a linear model, and continuous covariates as linear, exponential, and power models [22].

For the forward selection step a *p* < 0.01 (*χ* ^2^_*p*=0.01,*ν*=1_ = 6.63) was used while the backwards deletion step used a *p* < 0.001 (*χ* ^2^_p=0.001,*ν*=1_ = 10.83).

Body size using LBW was not included in the SCM procedure but was an a priori part of the structural model, and its effect on both apparent clearance (CL/F) and apparent volume (V/F) was incorporated using allometric equations.

### Model qualification

A visual predictive check (VPC) was performed on the final model parameter estimates to evaluate its predictive performance. The VPC looks at the model’s ability to simulate the same data that have been used for the model development [24]. Brivaracetam concentrations were simulated 1000 times using the same dose and covariate data and sampling times from the subjects that were in the data set. Additionally, model evaluation included the graphical evaluation of goodness of fit.

### Simulation approaches for paediatric dose adaptations

The obtained covariate model was used to assess whether dosing strategies for specific paediatric groups were adequate in producing brivaracetam concentration profiles consistent with the predicted adult concentration range. The population estimates from an adult pharmacokinetic model in patients from phase 3 efficacy trials [25] were used to derive the median and 90% of the predicted steady state (Css) levels for adults receiving 100 mg brivaracetam bid (i.e. the highest recommended dose), irrespective of AED co-administration. Paediatric simulations using the population estimates from the final paediatric model were performed with the study administration schedule (2.0 mg/kg bid for patients <8 years and 1.6 mg/kg bid with a maximum of 100 mg bid for patients ≥8 years). Additional schedules of 2.0 and 2.5 mg/kg bid were also investigated with a maximum dose of 100 mg bid, independent of age.

The developed population model scaled the pharmacokinetic parameters using LBW. In order to allow assessment of dosing-adequacy for WT and age, the NHANES DXA database [26] was used to provide linked demographic variables (age, WT and LBW) to drive the simulations on 11,087 children between 0 and 16 years, with WT ≤100 kg PB, CBZ and VPA co-administration status was randomly sampled from the paediatric study dataset. The results of the simulations were illustrated graphically by superimposing the distribution of simulated paediatric Css values (median and 90% prediction interval) on the adult reference range, together with the predicted Css values for the individual paediatric patients using the empirical Bayes estimates (EBEs) from the final model.

### Metabolites exposures

In order to obtain summary estimates of exposure for the three metabolites, a basic model with first-order formation rate, single compartment distribution, first-order elimination rate and scaling using LBW was applied to all metabolites separately to obtain CL estimates. These CL EBEs were used to estimate steady state concentrations (Css) of each metabolite at the maximum dose of the applied dosing regimen. Additionally, Css ratios of the hydroxy metabolite to the parent drug and to the sum of the four moieties were calculated, in the overall study population and in the subgroup of patients not taking inducing AEDs.

## Results

A total of 600 BRV plasma concentration-time records were available in 96 paediatric patients with a well-balanced distribution of patient numbers aged 1 month to <2 years, 2 to <6 years, 6 to <12 years and 12 to <16 years age groups of 29, 26, 24 and 17 patients respectively. Brivaracetam pharmacokinetics was adequately described using a one-compartment model with first-order absorption and allometric scaling of clearance and volume using body size. A combined proportional and additive residual error model yielded no improvement over a proportional residual error model. Implementing allometric scaling of brivaracetam clearance and volume resulted in a 204.9 point drop in OFV using theoretical allometric exponents and lean body weight (*p* < 0.0001). When WT was used instead of LBW, OFV increased by 11.42 points, and therefore the stepwise covariate search model development continued using LBW. In the first forward search step, the SCM procedure selected enzyme inducer AEDs co-administration as most significant covariate. No further covariates were detected, and the backward search step indicated that the inducer covariate must be retained in the model.

No effects of categorical covariates race, ethnicity, sex, CYP3A or CYP2C19 inhibitors were detected and no effects of age, PCA, or eGFR were detected using linear, exponential, or power relationships. This could conclude covariate model development. However, in the first step, both CBZ, PB, and VPA were highly significant covariates and the aggregate effect of the inducer covariate obscures the individual contributions. Therefore, individual contributions of these three AED effects were estimated instead of the inducer covariate to allow quantification of their respective effects.

All three AEDs had significant effects on CL on their own. In the combined estimation, co-administration of PB was estimated to induce a 40.8% increase in clearance, CBZ a 47.9% increase, and VPA a 10.1% *decrease*. Even though VPA did not meet the criteria for inclusion in the model, it was retained nevertheless since quantification of its contribution was considered informative. Population parameters for the final model are provided in Table 1. Shrinkage for both V/F and Ka was substantial (45.6 and 73.4%, resp.) indicating that the information on these parameters in the current data set is limited, but CL, determining exposure, was accurately estimated with low shrinkage (6.1%). High shrinkage in itself is not an issue, but limits diagnostics using individual empirical Bayes estimates [27].

**Table 1:** NONMEM parameter estimates (with 95% confidence intervals) and bootstrapped estimates (median and 95% of estimates) for the final paediatric brivaracetam model using LBW-dependent CL and V (normalised to a 50-kg LBW adult), and effects of co-administration of PB, CBZ and VPA

Parameter	NONMEM estimates	Bootstrapped estimates	IIV	Shrinkage (%)
CL/F (L/h)	3.63 (3.42/3.85)	3.62 (3.42/3.86)	22.8%	6.1%
V/F (L)	47.8 (43.1/52.5)	47.6 (43.1/51.7)	16.7%	45.6%
Ka (1/h)	1.84 (0.91/2.78)	1.83 (1.26/5.27)	31.9%	73.4%
Allometric scaling CL/F	0.750 fixed			
Allometric scaling V/F	1.00 fixed			
CL change with PB (%)	+40.8 (+19.9/+65.2)	+39.8 (+18.3/+65.8)		
CL change with CBZ (%)	+47.9 (+27.8/+71.2)	+48.2 (+26.2/+73.0)		
CL change with VPA (%)	−10.1 (−18.5/−0.8)	−10.0 (−19.5/−0.8)		
Residual error (CV, %)	23.4 (19.6/27.1)	23.2 (19.7/26.7)		9.2%

Goodness of fit plots using conditional weighted residuals demonstrated the absence of systematic model misspecification regarding time after dose, and absence of residual effects of WT, LBW and age (Supplemental Fig. 1). The smooth through the plot of absolute individual weighted residuals was almost horizontal indicating the applied residual error model was adequate (Supplemental Fig 2). Finally, goodness of fit plots of brivaracetam concentrations vs. population predictions and individual predictions demonstrated the absence of systematic model misspecification (Supplemental Fig 3).

Accuracy of final population parameter estimates was assessed by bootstrapping the paediatric dataset 1000 times and re-estimating the parameters for these datasets. Twenty-three non-successful runs were excluded from the calculations. The results are provided in Table 1 illustrating the close correspondence between NONMEM estimates and the bootstrapped result. The only real deviation is seen for the absorption rate constant (Ka) where the distribution of bootstrapped estimates is far more skewed than the symmetrical distribution assumed in NONMEM.

The VPCs that were performed for the final model were split by study occasion, age group, and AED co-administration status. For the overall population, split by study occasion, there was a close correspondence between 5th, 50th (median), and 95th percentiles of the observed data and corresponding simulated quantiles (see Supplemental Fig. 4). When the data were further split by age category, the ranges widened due to the reduced number of observations per category, but observed percentiles still tended to lie within the simulated areas (Supplemental Fig. 5). When the dataset was split by AED co-administration, again observed percentiles fell within the simulated ranges (Supplemental Fig. 6). These VPCs indicate that the final model is capable of simulating concentration profiles that correspond to the original data.

**Fig. 1:**
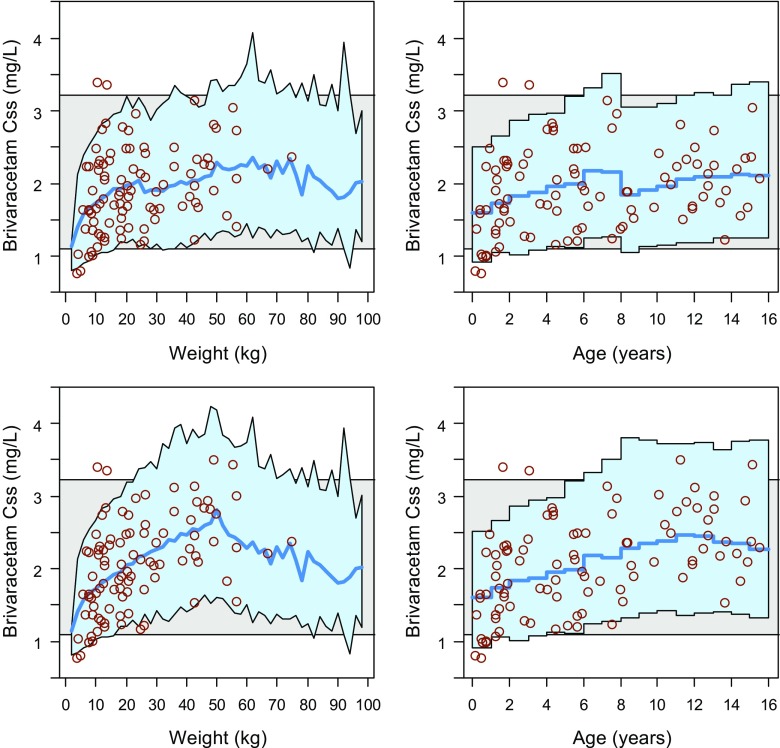
Predicted Css by weight (*left*) and age (*right*) using the final paediatric population PK model (*red circles*: individual predictions) for children from the NHANES database using 2 mg/kg bid for patients <8 years, and 1.6 mg/kg bid for patients ≥8 years with 100 mg bid maximum dose (*top*), or 2 mg/kg bid with 100 mg bid maximum dose for all patients (*bottom*). The *blue line* is the median simulated paediatric Css and the *blue shaded area* encompasses 90% of the simulated paediatric patients. The *horizontal grey band* encompasses 90% of the simulated adult patients receiving 100 mg bid

The simulated trial dosing scheme put most of the model-predicted concentrations (blue area) in the adult range (grey area) and individual predictions for N01263 paediatric patients (red dots) were mostly contained within the model-predicted range (see Fig. 1, top row). However, children aged <1 year and weighing <10 kg appeared to have a reduced exposure even though still mostly within the adult range.

**Fig. 2:**
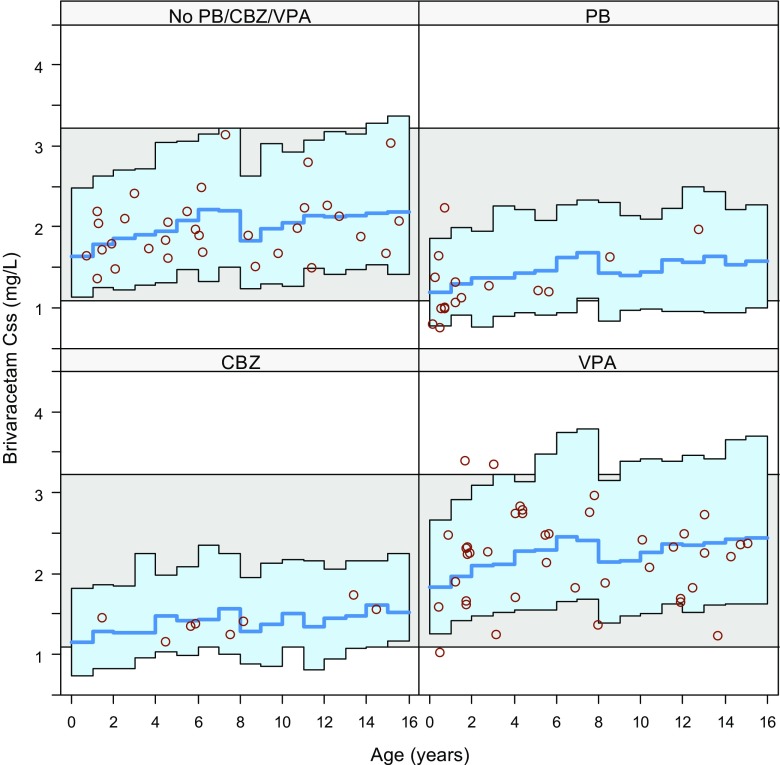
Predicted Css by age split by co-administration with PB, CBZ and VPA or absence of PB/CBZ/VPA using the final paediatric population PK model (*red circles*: individual predictions) for children from the NHANES database using 2 mg/kg bid for patients <8 years, and 1.6 mg/kg bid for patients ≥8 years with 100 mg bid maximum dose. The *blue line* is the median simulated paediatric Css and the *blue shaded area* encompasses 90% of the simulated paediatric patients. The *horizontal grey band* encompasses 90% of the simulated adult patients receiving 100 mg bid

By subdividing the simulations by AED co-administration (background therapy with PB, CBZ, VPA, or excluding these 3 AEDs), it became apparent that the lower plasma concentrations in the young age group could be attributed to the more frequent co-administration of PB (see Fig. 2).

By removing the 20% dose reduction in children aged ≥8 years and simply dosing all patients with 2.0 mg/kg bid with a maximum of 100 mg bid, the predicted concentration profile across the paediatric population was still mostly contained within the adult Css range (Fig. 1, bottom row). Increasing the dose to 2.5 mg/kg bid across the entire population raised the exposure in smaller/younger children closer to the center of the adult concentration range, but increased the likelihood of over-dosing in older children, except in the presence of inducers (Supplemental Fig. 7).

Regarding exposure to the metabolites, the predicted mean (SD) average plasma concentrations in the group of patients not taking enzyme inducers and receiving the maximum scheduled dose (*n* = 71) were 0.36 (0.15) mg-eq/L, 0.12 (0.05) mg-eq/L, and 0.035 (0.012) mg-eq/L for the hydroxy, acid, and hydroxyacid derivatives, respectively, compared to 2.10 (0.51) mg/L for brivaracetam. The hydroxy to parent ratio was 18.4 (10.8)%, while the ratio of hydroxy to total (sum of all 4 compounds) was 14.1 (6.7)%.

## Discussion and conclusions

A population PK model was developed for brivaracetam in paediatric patients consisting of first-order absorption, single compartment distribution, and first-order elimination components with allometric scaling of CL and V parameters using fixed theoretical allometric exponents. Lean body weight provided a slightly better description of CL differences between patients than WT. Differences between LBW- and WT-based allometric scaling were small because WT and LBW were very highly correlated for these paediatric patients.

The paediatric population clearance, 3.63 L/h, (normalised to a typical adult) was consistent with the population clearance value of 3.58 L/h found in adults with epilepsy [25] and with mean values of 3.37 and 3.64 L/h reported in two trials in healthy adults [13, 27]. Exposures to the three inactive metabolites appeared similar to those found in healthy adults, albeit the hydroxylation ratio was somewhat higher in children with epilepsy (18 vs 9% [28]), at least partly due to background therapy with enzyme-inducing AEDs.

Covariate analysis indicated that co-administration with PB, CBZ or VPA was associated with a clearance modification: co-administration with PB, or CBZ was associated with a 29.0 and 32.4% decrease in exposure, respectively. Co-administration with VPA was associated with an 11.2% increase in exposure. Given that the current data set only contained a single patient that received both PB and CBZ, no conclusions are warranted about the combined effect of CBZ and PB.

No effects on clearance could be attributed to race, ethnicity, sex, co-administration of CYP3A or CYP2C19 inhibitors, age, PCA or eGFR. The absence of a detectable CYP3A or CYP2C19 inhibitor effect is no evidence of absence of interaction since only two and seven patients were co-administered with a CYP3A (one each on fluconazole and clarithromycin) or CYP2C19 (all on omeprazole) inhibitor, respectively. It should be noted that covariate analysis only detects associations, and estimated effects should not be confused with causal relationships. The increased exposure observed in the presence of VPA for instance could be due to confounding with underlying unknown factors. VPA is a CYP2C9 inhibitor [29, 30] while BRV is not a substrate of this isoenzyme (only one of its metabolites is, and only partially). In addition, VPA did not increase BRV concentrations in adults, as evidenced in a population analysis around 20 times the size of the present paediatric population [25]. Further, BRV plasma protein binding is insignificant [11] which rules out the theoretical possibility of a displacement-based interaction [30]. However, a well-known side effect of chronic VPA is to increase body weight and fat significantly [31], which could have an influence on the pharmacokinetics of BRV, being a hydrophilic compound. Interestingly, levetiracetam exposure appeared 23% higher in a subgroup of adult patients co-administered with VPA; it was hypothesised that the effect could arise from the known association between VPA and increased body fat, since levetiracetam is negligibly metabolised by cytochrome P450 enzymes [32]. Age-related maturation of metabolism could not be detected. Increased clearance in young children could be associated with the more frequent co-administration of PB as compared to older children.

Simulations demonstrated that the majority of children reached an exposure similar to adults using the trial dosing regimen of 2.0 mg/kg bid for patients <8 years and 1.6 mg/kg bid with a maximum of 100 mg bid for patients ≥8 years, although smaller children tended to have a slightly reduced exposure, in the lower end of the adult range, when co-administered with enzyme-inducing AEDs.

Simulations suggested that a switch to an age-independent dosing regimen of 2.0 mg/kg bid with a maximum of 100 mg bid would result in profiles comparable to those attained with the weight and age-dependent trial dosing regimen.

## Electronic supplementary material

ESM 1 (DOCX 223 kb)
