# Supplementary material for: Brivaracetam population pharmacokinetics in children with epilepsy aged 1 month to 16 years
Source: Eur J Clin Pharmacol. 2017 Mar 9;73(6):727–33. doi: 10.1007/s00228-017-2230-6 (PMC5423986; doi:10.1007/s00228-017-2230-6)
Supplement: Supplementary file 1 — (DOCX 223 kb) [file 228_2017_2230_MOESM1_ESM.docx]

Online supplementary material for "Schoemaker R, Wade JR, Stockis A. Brivaracetam population pharmacokinetics in children with epilepsy aged 1 month to 16 years"

# Demographic summary table

Supplementary Table 1 Overall summary of demographics, co-medication intake, race/ethnicity, sex and age category

| Demographics | Mean | SD | Median | Min | Max | N |
| --- | --- | --- | --- | --- | --- | --- |
| WT (kg) | 24.2 | 16.17 | 18.9 | 3.9 | 75.0 | 96 |
| LBW (kg) | 20.0 | 12.42 | 16.8 | 3.6 | 53.8 | 96 |
| eGFR (mL/min/1.73m^2^) | 114.4 | 33.05 | 112.6 | 49.0 | 218.1 | 96 |
| Age (yrs) | 6.2 | 4.74 | 5.5 | 0.2 | 15.6 | 96 |
| *Co-medication* | *N* | *%* of total number of patients | | |  |  |
| Carbamazepine | 9 | 9.4 |  |  |  |  |
| Phenytoin | 1 | 1.0 |  |  |  |  |
| Phenobarbital or primidone | 16 | 16.7 |  |  |  |  |
| Valproate | 49 | 51.0 |  |  |  |  |
| CYP3A inhibitor † | 2 | 2.1 |  |  |  |  |
| CYP2C19 inhibitor ‡ | 7 | 7.3 |  |  |  |  |
| *Race/ethnicity* | *N* | *%* of total number of patients | | |  |  |
| Caucasian | 77 | 80.2 |  |  |  |  |
| Black | 4 | 4.2 |  |  |  |  |
| Other | 15 | 15.6 |  |  |  |  |
| Hispanic or latino | 18 | 18.8 |  |  |  |  |
| *Sex* | *N* | *%* of total number of patients | | |  |  |
| Boys | 47 | 49.0 |  |  |  |  |
| Girls | 49 | 51.0 |  |  |  |  |
| *Age category* | *N* | *%* of total number of patients | | |  |  |
| 1 month - <2 yrs. | 29 | 30.2 |  |  |  |  |
| 2 yrs. - <6 yrs. | 26 | 27.1 |  |  |  |  |
| 6 yrs. - <12 yrs. | 24 | 25.0 |  |  |  |  |
| 12 yrs. - <16 yrs. | 17 | 17.7 |  |  |  |  |

†clarythromycin (n=1), fluconazole(n=1)

‡omeprazole(n=7)

# Bioanalytical method

The plasma concentrations of brivaracetam and its 3 metabolites (acid, hydroxy, and hydroxyacid) were determined in 600 samples obtained from 96 subjects on active treatment. The validated bioanalytical method used solid phase extraction followed by liquid chromatography with triple quadrupole mass spectrometry detection using a previously described method with minor adaptations [12]. Briefly, plasma aliquots (50 µL) were mixed with internal standards (50 µL of 50 ng/mL d6-labelled brivaracetam and d6-labelled metabolites in water) and 400 µL of 0.1% trifluoroacetic acid (TFA) in water (pH 2.5), and were vortex-mixed. The samples were submitted to a solid-phase extraction procedure on preconditioned Sep-Pack tC18 cartridges (Waters, Milford, MA, U.S.A.). After washing twice with 0.1% TFA in water (pH 2.5), the analytes were eluted with 1 ml acetonitrile and evaporated to dryness. After reconstitution in mobile phase A, the extracts were separated by gradient elution using 0.1% (pH 3.2) TFA in water-acetonitrile 99:1 v/v (phase A) and 0.1% (pH 3.2) TFA in water-acetonitrile 1:99 v/v (phase B) on a 150x2.1 mm ACE 3 C18-AE column with a 10x2.1mm guard column (Advanced Chromatography Technologies Ltd, Aberdeen, Scotland, UK). The chromatography system was a 1100 series HPLC system (Agilent Technologies Inc, Santa Clara, CA, USA) operated at a flow rate of 0.4 mL/min and an oven temperature of 50°C.

The mass spectrometer (Quattro Ultima, Waters Corp, Milford, MA, U.S.A.) was operated with an electrospray ionization (ESI) interface in positive MRM mode, and the monitored Q1–Q3 transitions were 213-168, 214-168, 229-184, and 230-184, for brivaracetam, and for the acid, hydroxy and hydroxyacid metabolites, respectively; the same transitions, shifted by 6 mass units, were monitored for the respective hexadeuterated internal standards. The quantification ranges were from 0.002 to 2.0 mg/L for brivaracetam and the hydroxyacid metabolite, and from 0.002 to 0.5 mg/L for the acid and hydroxyacid metabolites, respectively. Concentrations below the lower limit of quantification were discarded from the analysis.

Quality control plasma samples at nominal brivaracetam concentrations of 6, 75, and 1600 ng/mL yielded mean recovery errors of +4.2%, +6.1%, and -3.3% and precisions (RSD) of 9.3%, 3.0%, and 2.7%, respectively (n = 38). Similar recovery and precision values were obtained for the 3 metabolites.

# Goodness of fit plots (GOFs) final model

Supplemental Fig. 1 Brivaracetam goodness of fit plots using CWRESI for the final model

The black lines are zero lines, the blue lines are smoothes through the data. Conditional weighted residuals vs time after dose (TAD), WT, LBW and age. The darkness of the hexagons corresponds to the data density at that location.

Supplemental Fig. 2 Brivaracetam goodness of fit plots for the final model

The black lines (top) are zero lines, the blue lines are smoothes through the data. The black lines (bottom) are the normal curves generated using the calculated mean and standard deviation of the residuals. The darkness of the hexagons corresponds to the data density at that location.

Supplemental Fig. 3 Brivaracetam goodness of fit plots for the final model

The black lines are lines of identity, the blue lines are smoothes through the data. The darkness of the hexagons corresponds to the data density at that location.

# Visual predictive checks (VPCs) final model

Supplemental Fig. 4 Visual Predictive Checks for brivaracetam time profiles for the final model

Left: linear y axis, right: logarithmic y axis. Red lines are the 5^th^ , 50^th^ (median) and 95^th^ percentiles of the observed data and the light blue areas contain 90% of the simulated corresponding quantiles.

Supplemental Fig. 5 Visual Predictive Checks for brivaracetam time profiles by age category for the final model

Left: linear y axis, right: logarithmic y axis. Red lines are the 5^th^ , 50^th^ (median) and 95^th^ percentiles of the observed data and the light blue areas contain 90% of the simulated corresponding quantiles.

Supplemental Fig. 6 Visual Predictive Checks for brivaracetam time profiles by AED category for the final model

Left: linear y axis, right: logarithmic y axis. Red lines are the 5^th^ , 50^th^ (median) and 95^th^ percentiles of the observed data and the light blue areas contain 90% of the simulated corresponding quantiles.

Supplemental Fig. 7 Predicted Css by age split by co‑administration with PB, CBZ and VPA or absence of PB/CBZ/VPA using the final paediatric population PK model (red circles: individual predictions) and predicted ranges for children from the NHANES database using 2.5 mg/kg bid for all patients with 100 mg bid maximum dose.

The blue shaded area encompasses 90% of the simulated paediatric patients, the blue line is the median simulated paediatric Css. The horizontal grey bar is the predicted 90% CI Css of the adults receiving 100 mg bid.
